# Iatrogenic harm in functional neurological disorder

**DOI:** 10.1093/brain/awae283

**Published:** 2024-09-06

**Authors:** Caoimhe Mcloughlin, Wei Hao Lee, Alan Carson, Jon Stone

**Affiliations:** Centre for Clinical Brain Sciences, University of Edinburgh, Edinburgh Bioquarter, Edinburgh E16 4SB, Scotland, UK; Centre for Clinical Brain Sciences, University of Edinburgh, Edinburgh Bioquarter, Edinburgh E16 4SB, Scotland, UK; Centre for Clinical Brain Sciences, University of Edinburgh, Edinburgh Bioquarter, Edinburgh E16 4SB, Scotland, UK; Centre for Clinical Brain Sciences, University of Edinburgh, Edinburgh Bioquarter, Edinburgh E16 4SB, Scotland, UK

**Keywords:** functional neurological disorder, conversion disorder, iatrogenic harm, misdiagnosis, stigma

## Abstract

Functional neurological disorder (FND) is continuing to gain increasing recognition globally as a valid and potentially treatable disorder. Iatrogenic harm towards patients with FND is significant, however, and has been around for centuries. Despite advances in our understanding around the aetiology, pathophysiology and treatment of FND, many aspects of such harm continue to persist. Avoidance of iatrogenic harm has been highlighted by clinicians as one of the most important therapeutic considerations in FND; however, the sources and range of potential harms, or indeed ways to mitigate them, have not previously been summarized.

Using a combination of clinical and research experience and scoping review methodology, this review aims to describe the main sources of iatrogenic harm towards patients with FND, including harm from misdiagnosis, delayed diagnosis and treatment, direct harm from professional interactions, other stigma-related harms, harm related to diagnostic overshadowing and over-diagnosis of FND. We also describe some potential ways to address and prevent such harms, such as ways to reduce misdiagnosis with a focus on rule in signs, optimizing teaching and communication, ensuring parity of FND with other medical conditions and continued integration of patient and professional organizations.

## Teaser

Functional neurological disorder is a common and treatable condition at the interface of neurology and psychiatry. Mcloughlin *et al.* outline some of the ways in which patients have suffered and continue to suffer iatrogenic harm due to structural neglect of this condition, and consider potential ways to address such harms.

## Introduction

‘I will use treatment to help the sick according to my ability and judgment, but never with a view to injury and wrong-doing’.—Translation of Hippocratic Oath.^1^

Functional neurological disorder (FND) is now recognized as a common, valid and treatable disorder,^2^ with population studies suggesting a prevalence of 50–100 cases in 100 000.^2^ FND typically affects women, with a female preponderance of 60%–80% across studies, although the gender gap narrows with increasing age.^3^ It remains, however a neglected condition, influenced by outdated misperceptions and attitudes, and inadequate knowledge and training.^4-6^ Although newer developments aim to address this, (for example the FND Society and patient-led FND charities), the consequences of this systemic neglect are far-reaching and continue to cause harm to patients with FND.

Iatrogenic harm refers to harm experienced by patients resulting from medical care and is a concept that will be familiar to every practicing physician. We acknowledge that iatrogenic harm is a complex issue, can be difficult to avoid and often occurs in the context of intended broader benefit to the patient—the side effects of chemotherapy or antibiotics for example. Some of these harms are unavoidable, an inevitable consequence of the advance in medical treatment—so-called ‘diseases of medical progress’.^7^ It is hoped in most cases that treatment is given in the knowledge of weighing up potential risks and benefits. With FND however, there have been centuries of harm towards patients that has persisted despite patients and professionals highlighting such issues and ways to alleviate them. The repercussions of such harm span a wide spectrum and are driven by historical inaccuracies, failure to recognize FND as a valid condition and indifference in training and service development.

A large survey of neurologists from 92 countries showed that 58% of clinicians considered avoidance of iatrogenic harm the most important therapeutic action in FND,^8^ but the range of potential harms has not previously been summarized. This paper aims to review eight main sources of such iatrogenic harm related to FND and considers potential ways to address such harms (Tables 1 and 2).

**Table 1:** Eight sources of iatrogenic harm in functional neurological disorder (FND)

Sources of harm	Example
Unnecessary medical treatment from misdiagnosis of FND as another condition	Misdiagnosis of FND as epilepsy can cause harm from unnecessary (often teratogenic) medications, and, in cases of presumed ‘status’, harm can occur as a result of unnecessary intubation and it’s sequelae.
Psychosocial harm from misdiagnosis of FND as another condition	Having a diagnosis changed—for example from multiple sclerosis to FND—can have psychological and social repercussions, such as impacting on identity, inducing feelings of shame or unjustifiable responsibility for symptoms, or influencing relationships with caregivers and colleagues.
Harm from delayed diagnosis and treatment	Patients often wait much longer for a diagnosis of FND compared with other conditions, delaying access to treatment and potential recovery.
Direct harm and assault in medical settings	There are reports of patients with FND being ridiculed, unnecessarily pierced with needles, or being pushed forcefully in wheelchairs in an effort to stop their functional seizures.
Psychological harm through the stigma of being labelled, dismissed and doubted	Terms such as ‘pseudo’ have led clinicians to associate FND symptoms with ‘fake’, and patients have been accused of faking their symptoms for attention. Studies from the perspective of both patients and clinicians outline how patients can be treated in dismissive, rejecting and derogatory ways when seeking help.
Misuse and misinterpretation of the concept of a functional disorder	The term functional disorder (including FND) is often misunderstood and has widely and inaccurately been depicted as a synonym for clinicians meaning ‘it’s not real’ or ‘dustbin diagnosis’ or ‘you’re imagining it’. This leads to doubt and worry when patients are researching their condition, particularly online.
Diagnostic overshadowing: over-interpretation of investigations and under-investigation in established FND	Over-interpretation of common signs such as ‘normal for age’ white matter change or degenerative changes on MRI may be given undue weight when FND is the actual diagnosis. Conversely, harm can also occur when patients with FND develop new signs that are not investigated properly, as clinicians do not look past the patient’s existing FND for a cause.
Harm related to misdiagnosis of other conditions as FND and overdiagnosis of FND	Premature or misdiagnosis of FND, based on, for example, an unusual presentation or a psychiatric comorbidity, without an adequate period of assessment or investigation can lead to harm. In addition, the use of an FND ‘disorder’ diagnosis may not be appropriate for someone with mild symptoms or where a more specific term, such as functional cognitive symptoms, may be better.

**Table 2:** Five potential ways to address and mitigate harm

1. Reduce the risk of misdiagnosis: make FND a diagnosis of inclusion
2. Introduce FND early in the differential and contextualize investigations
3. Teaching: focus on communication and stigma
4. Parity for FND in service development and research
5. Ongoing promotion and integration of patient and professional organizations

FND = functional neurological disorder.

## Literature search

We used a combination of scoping review methodology and clinical and research knowledge to inform our search and construction of themes. The process of preparing a recent review on the topic of stigma in FND^9^ led us to recognize that iatrogenic harm in FND is a topic that has not been collated and described in an organized way in the literature. Informed by some of the themes from this systematic review, and using the expertise of the two senior authors (J.C., A.C.), who have been working clinically and conducting research in the field of FND for over 25 years, we developed core themes that we considered relevant to iatrogenic harm, namely: misdiagnosis, delayed diagnosis, direct harm/assault and stigma. These themes were expanded upon in detail by the authors (C.M. and W.L.), who conducted a scoping review of these topics on the main databases: Medline, PsycInfo, Embase and Google Scholar. This search was conducted in line with scoping review methodology in accordance with the five-step framework proposed by Arksey and O’Malley.^10^ We decided on a scoping review because it allows for a broad literature search and for the organization of a heterogeneous literature base. This process resulted in themes related to harm sources 1–5, outlined in Table 1.

After conducting this scoping review of the initial five themes, we realized that there are further sources of harm that were not easily found or described in the literature, which informed the remainder of the sources of harm (bottom three sources in Table 1). We wrote about these, and the mitigating factors (Table 2), from a mixture of our clinical and research experience, augmenting with current relevant literature from the area.

## Unnecessary treatment from misdiagnosis of FND as another condition

Regarding misdiagnosis, this is not unique to FND and is an inevitable part of practicing as a clinician. We acknowledge the harm experienced by patients described in the following scenarios is at times unavoidable and arises in the context of well-meaning attempts to treat and prevent illness.

FND should be diagnosed by inclusion, and not by ‘ruling out’ other explanations. However, there remain many who are concerned about missing a diagnosis of another neurological condition such as epilepsy, stroke or multiple sclerosis (MS), which might be perceived to be a larger clinical error than missing FND. Any misdiagnosis is likely to cause harm to some extent. However, evidence shows that misdiagnosis of another neurological condition as FND occurs no more commonly than the baseline for many neurological conditions.^11-13^ Misdiagnosis of someone who really does have FND as another neurological condition, may even be more common than the other way round^14^ and certainly appears to be the case for epilepsy and functional seizures.^15^ It is important to note the question of misdiagnosis is not a binary one, since FND is also commonly comorbid with other neurological conditions such as MS, Parkinson’s disease and epilepsy.^13,16^ FND can occur subsequently, as commonly occurs with epilepsy,^17^ simultaneously, or, in the prodrome, as occurs in Parkinson’s disease.^18,19^

### Misdiagnosis of functional seizures as epilepsy

‘But for some or other reason the pills made me sicker, not better … I think the medication was the worst…because it makes you feel really clumsy and confused’—Patient with functional seizures, South Africa.^20^

The frequency of ‘false positive’ diagnoses of epilepsy has been reported to range from 2%–71%, with functional seizures (also called dissociative or non-epileptic seizures) being one of the most common final diagnoses.^21^ There is substantial evidence in the literature describing patients misdiagnosed in ‘status epilepticus’ when they were in fact having prolonged functional seizures.^15^

Many have been administered repeated doses of anti-epileptic medications in the emergency setting, often on multiple occasions, including children.^22-24^ Other sequelae from this misdiagnosis include unnecessary invasive major vessel venous access, Intensive Care Unit (ICU) admission with assisted ventilation/intubation and even anti-convulsant induced respiratory arrest.^25-28^ One study evaluated 80 patients admitted via the emergency department with a diagnosis of functional seizures without comorbid epilepsy, finding that 15% of these patients underwent intubation, with 65% (*n* = 31) of the non-intubated patients receiving anti-epileptic mediations in the emergency department (usually intravenous benzodiazepines).^28^ Several of these patients had prolonged length of inpatient stays and went on to develop further iatrogenic complications such as hypotension, requiring vasopressors, and nosocomial infection. A more recent study evaluated 980 inpatients aged eight and above, finding that 8.1% (*n* = 79) of patients initially thought to have status epilepticus had a final diagnosis of functional seizures, and this risk was highest in children and young adults. Furthermore, respiratory intubation and ICU admission were documented in a substantial portion of this group.^29^ The above studies show that vulnerable populations such as those with learning disability and children appear particularly at risk from this misdiagnosis.

In addition to ‘status’, there are less acute but nonetheless harmful implications of being misdiagnosed as having epilepsy. For example, there are reports of patients, including women and young children, remaining on potentially teratogenic and toxic medications in the longer term, even after the diagnosis of epilepsy was retracted, in the absence of another clear indication such as bipolar affective disorder or chronic pain.^30,31^ In a study of 288 patients with functional seizures, 154 (53%) patients were being treated with anti-epileptic medications, despite a much smaller number (*n* = 32/11%) having comorbid epilepsy.^32^

A small proportion of patients are likely prescribed anti-epileptic medications for other indications such as mood stabilization or pain—although these scenarios would be rare in children. Such regimes may perpetuate further confusion and harm.^33^ Furthermore, one study found that 13 patients were implanted with a vagal nerve stimulator for ‘intractable seizures’ later found to be exclusively functional seizures, diagnosed with video-EEG.^34^

An important cohort study showed the mortality rate in patients with functional seizures is 2.5 times higher than the general population and at a risk comparable to drug-resistant epilepsy.^35^ A further Danish study found that mortality was three times higher in patients with functional seizures compared with matched controls.^36^ It is important to recognize there are many potential reasons for this—such as issues related to physical and psychiatric comorbidities, including suicide.^37^ However, there is suggestion among experts that iatrogenic harm from the misdiagnosis of epilepsy may be an important contributory factor to mortality.^29,38^

Concerningly, antiepileptic medications have been used in suicide attempts in children who were referred to a tertiary centre for epilepsy treatment and diagnosed with functional seizures.^39^ Elderly patients are also at particular risk of unnecessary iatrogenic harm given the increased propensity for falls and delirium with antiepileptic medications, as a recent case in the literature reported.^40^

We recognize these risks are not unique to FND and epilepsy. Suicide attempts can occur with any medication, whether or not it is prescribed inappropriately, and elderly patients are more likely to suffer the risks associated with side effects and polypharmacy in general. However, prescribing CNS agents that have high potential for lethality, falls, cognitive difficulties and subsequent complications in the absence of a clear indication for their use carries a particularly high potential for harm in these more vulnerable groups. We do also recognize however, that there are cases where a diagnosis is difficult, and the risks of untreated epileptic seizures may outweigh the risk of a trial of medication while ongoing efforts occur to clarify a diagnosis.

### Misdiagnosis of FND as stroke, multiple sclerosis, myasthenia, autoimmune encephalitis and dementia

Functional motor symptoms such as weakness and tremor are among the most common types of FND presentation,^2^ but these can also go misdiagnosed as other neurological conditions such as stroke, Parkinson’s disease or MS, with iatrogenic harm complications. A large-scale study spanning 43 countries showed that FND has shown to be a common cause of a ‘stroke mimic’ presentation given intravenous thrombolysis.^41^ Thrombolysis appears to be a low-risk intervention in people without acute stroke and carries considerable potential benefit so therefore this is a calculated risk. Nonetheless, it is one that could be mitigated even more with optimal knowledge of the distinguishing semiological features of FND.^42^

In another study of 320 people with motor FND, 238 (74%) received one or more initial misdiagnoses of different neurological diseases such as Parkinson’s disease, cerebrovascular disease, essential tremor, epilepsy or neuroinflammatory disease, with a diagnostic delay of about 6 years. Importantly, during this period of misdiagnosis, these patients were not being offered the correct services, delaying treatment.^19,43^ There have also been case reports of deep brain stimulation mistakenly given for FND, with potential adverse effects.^44^

A survey of 122 North American MS specialists found that FND, fibromyalgia and other related disorders were common reasons for misdiagnosis among an estimated 598 patients, with around half of those taking a disease modifying therapy.^45^ Another study of 110 patients misdiagnosed with MS recorded that 12 (11%) patients had FND and another 16 had fibromyalgia; 70% were given disease modifying therapy, 21 of these for over 10 years.^46^ A study of eight people misdiagnosed with myasthenia gravis found that four had FND and were exposed to unnecessary surgery and immunosuppression.^47^ FND was the commonest final diagnosis (25%) in another study of 107 people misdiagnosed with autoimmune encephalitis, with exposure to heavy duty immunosuppression.^48^

Functional cognitive symptoms are characterized through their typical clinical features, and people with these symptoms are sometimes misdiagnosed as dementia, given the high rate of these presentations to memory clinic.^49^ There is potential in such scenarios that inaccurate treatments are offered, in addition to the potential psychosocial harm of a dementia diagnosis, although data on treatments in this group are lacking.^50^

## Psychosocial harm from misdiagnosis of FND as another condition

‘[I] felt suicidal and unprepared for this diagnosis. All at once a lot of significant information that had affected my career prospects and decisions not to have children was given to me. I felt I had been cheated and wanted my life back again’—Patient with a diagnostic change from epilepsy to functional seizures.^51^

There are psychological and social repercussions from misdiagnosis of FND as another condition. Many patients have reported feelings of confusion, anger, uncertainty and mistrust when their diagnoses were changed from epilepsy to functional seizures.^51-53^ For many, the unique identity associated with having that illness may have formed a big part of their life, such as belonging to a patient group or charity. It may also have impacted people’s decisions around starting a family, or career prospects.^51^

Having to tell people that you no longer have epilepsy or MS may feel embarrassing or hard to explain, especially when it is hard for people to communicate the diagnosis of FND to family, friends or work.^53^ Neurologists have reported reluctance to ‘undiagnose’ MS, and the role of family members and practical issues like welfare benefits may make it even more difficult.^45,54^ Being mislabelled can affect how others perceive an individual also—one study examining paediatric coding of functional seizures found that 17 different diagnostic International Classification of Diseases (ICD)-10 codes were used by 61 paediatricians and, somewhat bizarrely, the most common code fell under ‘F91.8: Other Conduct Disorders’.^55^ This is likely to have significant consequences on how these children are perceived both in and outside clinical settings, given how Conduct Disorder manifests with anti-social and aggressive characteristics and deceitfulness.^56^ On this point of coding, a US study demonstrated that neurologists, after diagnosing FND, selected FND-related ICD-10 codes in only 22.8% of consultations. The biggest predictor of non-coding was the belief that FND was a ‘diagnosis of exclusion’.^57^

In addition, there is self-stigma that comes with the ‘status loss’ of not having a more well-known neurological illness. Studies have reported feelings of shame and self-blame when diagnoses were changed, as if the situation was now somehow the individual’s fault.^51-53,58^ On a more practical social note, they may have lived with the influence of restrictions imposed by the wrong diagnosis for several years, having had unnecessary driving restrictions or other social and lifestyle constraints.

There are identity issues with being labelled with FND also, especially after a previous more socially acceptable, credible diagnosis and this likely affects treatment engagement.^51,52,59,60^ This self-stigma may lead to some not disclosing their true diagnosis^61^ or pretending they do not have FND, maintaining they had ‘epilepsy’^62^ or ‘brain injury’.^63^

## Iatrogenic harm from delayed diagnosis and treatment

‘You just don’t fit into the little tick box that that you need to be in, I guess it’s funding and it’s so frustrating, from both sides, really frustrating. One of the things about FMD is that it’s just so isolating I found the whole NHS system really isolates people … there was just every door closed, you know, you don’t tick any the boxes so you can’t use their services so it’s so demoralising when that happens’—Patient with functional motor disorder, UK.^64^

The wait time for a diagnosis of FND has been shown to be particularly long compared with other neurological conditions.^65-67^ A study comparing diagnostic delay for patients with FND with other neurological disorders showed a longer diagnostic delay in the FND group compared with the other neurological disorders group (median = 48 months versus median = 12 months), with diagnostic delay correlating significantly with total costs in the entire sample, more strongly in the group with FND.^65^ In a service evaluation study comparing MS and FND, those with FND reported significantly longer waiting times for diagnosis and specialist care.^66^ Furthermore, in this study, significantly more reported that they felt they were not treated with respect and dignity, compared with almost no patients with MS feeling they were not treated with dignity and respect by professionals.^66^ A significant issue that often co-occurs with this is that with many patients remain on incorrect, harmful treatments whilst awaiting confirmation of their diagnosis,^68-71^ coupled with the added harm of not getting treatment for the correct diagnosis.

For functional seizures, the mean time from symptom onset to diagnosis in published studies ranges from 3 years to nearly 9 years.^69,72,73^ Some factors associated with delay in diagnosis include being female^74^; being prescribed anti-seizure medication^68,70,71^; and history of physical abuse^71^; and in children, a history of psychological abuse.^39^ These delays have been described as sources of distress and dissatisfaction^62,75^ and, as health professionals describe, are ‘costly and time consuming’.^76^

Taking a broader view, the lack of available treatment in many areas causes harm—a global problem that is worse in low-income countries.^77^ A study of healthcare professionals from 92 countries revealed that 48% of respondents indicated that low availability of services to refer to was one of the factors considered as ‘often’ or ‘always’ limiting patient management.^8^ In a report of 360 healthcare professionals from 17 countries, only one-third stated they follow-up patients with functional seizures.^78^ A survey of professionals from 63 countries revealed that ‘stigma/lack of awareness’ was the main barrier to the diagnosis and treatment for functional seizures in 70% of countries.^77^ This lack of follow-up translates to patients feeling rejected and dumped^79-82^ and is likely to affect clinical outcomes. A 14-year study looking at the long-term prognosis for patients with FND causing limb weakness showed that 80% still had the symptom at follow-up, with outcomes worse for those with a long duration of symptoms and better for those with early diagnosis and treatment.^83^

It is important to note that delayed diagnosis of FND is not the case everywhere, and the tide is changing in many institutions. However, this discomfort and reluctance to manage this group means patients are unable to access clinical services—with FND itself reported as an ‘exclusion criterion’ in many services.^84,85^ This has a direct impact on the future generation of health professionals who can develop an interest in and treat FND.

## Direct harm and assault in medical settings

‘She then bent a pillow around my face, again to try and get a response … put me in a wheelchair with force and started shouting at me and pushing my shoulder and head back into the chair. I was very woozy and didn't understand what was happening’—Patient with functional seizures, UK.^86^

Some publications describe disturbing episodes of maltreatment of people with FND, often in emergency settings. Some examples include being unnecessarily pierced with needles, covered with a pillow or pushed violently in a wheelchair.^86^ One study reported a perception of being deliberately hurt during treatment with physiotherapy.^64^ Some harm may have been driven by cultural perceptions of illness; ‘when I went to the emergency room, for example, the nurses grabbed me, they told my mother to go to an exorcist. Those things hurt me’.^87^ Some tests described for FND involve potential injury to patients. The ‘arm drop test’ involves dropping a weak arm over a patient's face to see if they hit themselves. It has been suggested that people with FND do not hit themselves if they have limb weakness or are in the middle of a seizure, whereas people with other causes of weakness or epilepsy do. A test designed to allow a patient to hit themself is in our view unethical and has been described as unreliable.^88^ Assessing response to painful stimulation can be a legitimate part of the assessment of a comatose patient, but we are aware of it being performed in a needlessly harmful, arguably vindictive way in people with functional seizures.

Deceptive strategies—such as telling people with FND undergoing rehabilitation falsehoods such as: ‘failure to recover was definitive proof of a psychiatric aetiology’^89^—have intermittently been promoted as part of the treatment of FND, in a way that is at variance with usual healthcare. The arguments made for this, similar to the use of deceptive placebo in the diagnosis of functional seizures, is that deception is a lesser harm than not benefiting from treatment or being admitted to intensive care for seizures. However, honesty is a core and paramount component of a health professional relationship with a patient, and deception like this violates patient autonomy.^90^ In addition and importantly, such deceptive practices have not been proven to achieve better outcomes compared with practices using a transparent approach.

Anecdotally, the authors continue to hear of people being injected with large boluses of unnecessary intravenous saline and still having their arms dropped on them, particularly in emergency care settings. Jokes by health professionals at the expense of patients with FND have frequently been observed anecdotally and also reported in the literature.^91^

## Psychological harm through the stigma of being labelled, dismissed and doubted

### Labelling

‘Up to that time, hysteria had been the “bête noire” of medicine. The poor hysterics, who in earlier centuries had been burnt or exorcized, were only subjected, in recent, enlightened times, to the curse of ridicule; their states were judged unworthy of clinical observation, as being simulation and exaggerations’.—Sigmund Freud 1888.^92^

‘Labelling’ patients is a way of ‘categorizing’ an individual and usually has an intended meaning above and beyond the term. There is evidence that labelling leads to medical stereotyping and harm from healthcare rejection.^93,94^ For FND, terms like ‘hysteria’ and ‘pseudo’ have negative connotations, as well as likely reinforcing stereotypes about individuals with FND being non-genuine or out of control. Despite clear evidence of prejudice and use of the label hysteria to dismiss patients, especially women, the term was only removed from the Diagnostic and Statistical Manual of Mental Disorders (DSM)-III and from ICD-9 in the 1980s but has been continued to be used in research papers.

Other terms are not only misleading but have shown to be perceived as offensive and linked to treatment expectation.^95-97^ In a study of a healthy population of 87 adults ranking their preferred terms, pseudoseizures, conversion disorder and hysteria were the three least preferred terms. These three terms were also the most offensive (with functional non-epileptic attacks the most preferred term), and interestingly in this study, the terms pseudoseizures, psychogenic seizures and hysteria linked with expectations of non-recovery from treatment.^95^ In a mixed method study exploring this issue (39 patients), pseudoseizures and hysteria were the least preferred terms.^96^ Although it is less mainstream now, ‘pseudo’ continues to be used,^98,99^ perpetuating the myth that FND is fake and imagined, despite clear evidence to support the contrary.^100^ The term ‘psychosomatic’, historically associated with FND, originally meant to describe bidirectional disorders of mind and body, but its use has become problematic and stigmatizing for patients.^101^ This mode of usage has even extended to a recent Lancet editorial, where the author implies that ‘psychosomatic’ conditions should not be taken seriously.

‘long COVID is often easily dismissed as a psychosomatic condition. Given what we now know about the effects of long COVID and its biological basis, it must be taken seriously.’^102^

Moreover, the multitude of names that exist for FND is likely to add confusion during clinical communication or when individuals with FND or their caregivers are seeking information about the condition.

### Dismissal

‘People’s attitudes towards these patients is often really negative and dismissive, … not really giving them the time of day, being annoyed that they’re there really, if I can be frank, and just wanting them out’—Speech and language therapist, UK.^103^

The literature is rife with several examples of how many healthcare professionals still consider these patients the ‘“bête noire” of medicine’. Dismissal and prejudice towards patients range across a spectrum of mild disregard to more overt rejection and derision.^79-81,104,105^ Emergency care services, often the first port of call for patients with worrying symptoms have been described as more outright and explicit in their dismissal.^86,106^ A large survey of healthcare professionals from 92 countries showed that 29% reported somewhat or very much disliking seeing patients with FND.^8^ In an Australian study, 39% of healthcare professionals (*n* = 516) agreed they found patients ‘demanding and difficult to deal with’.^6^ In an Israeli study, 62% agreed that these patients arouse anger among staff, 60% agreed this group were treated with disrespect and 50% agreed patients misuse medical resources, with no correlation with professional speciality or seniority.^107^ One might argue that dismissive attitudes do not necessarily equate to iatrogenic harm, however, these attitudes have been shown to affect treatment.

In a recent study examining professional attitudes of ‘legitimacy’ towards FND and MS, professionals displayed stronger explicit attitudes that FND is illegitimate (doctors more than psychologists), with less favourable attitudes negatively associated with referral to physiotherapy.^108^ Patients and professionals have noted that anticipated stigma from professionals impacts motivation to seek help.^76,79^ It has been noted that patients are not received well in outpatient clinics: ‘nobody wants to deal with them’.^109^ Furthermore, stigma impacts on wellbeing and has been shown to correlate with patient quality of life in FND.^110,111^

### Doubted: feigning and voluntary control

‘Doctors in general … have been dismissive, rude and talked about me as if I wasn't there … I heard the paramedics discussing … “Yeah, I don't want to explain how you lost [the medication] and she’s faking anyway.” From there I don't recall exact words, but they went on to degrade me as a person. They were wheeling me into the ER as they were continuing their derogatory conversation, the ER staff joined in’—Patient with functional seizures.^86^

There are several reports outlining how healthcare professionals across every relevant discipline consider at least some patients with FND to be feigning or have voluntary control over their symptoms.^112-116^ A range of clinical and neurophysiological data indicates why feigning is rare and not a good explanation for FND.^100^

In a survey of 159 and emergency physicians and GPs, 38% believed that functional seizures were ‘voluntarily induced (patients are fakers)’, with emergency physicians more likely to consider this.^117^ One study of speech and language therapists revealed some clinicians thought malingering and functional stroke were interchangeable terms, with patients again being called ‘fakers’.^103^ In a recent survey of 152 psychiatrists, (34%) agreed that they were ‘often worried patients were actually malingering/faking/feigning’.^118^ Studies examining differences in illness perceptions indicate that doctors perceive patients to have more voluntary control over their symptoms in FND than those with epilepsy^119,120^—the latter study finding emergency care staff more likely to attribute functional seizures to ‘alcohol or behavioral issues’. These misperceptions were quite prominent in the paediatric realm also.^121,122^

From the patient perspective, this obviously has harmful repercussions. Patients are aware that other people think they are faking their seizures,^52,80^ whether it was felt implicitly or more explicitly being told they were making it up.^20,60,81,87^ It is likely that such patients will be less likely to seek help for troubling symptoms, leading to a worse prognosis. It may also impact on self-stigma, confidence and trust in others. These harmful interactions have led to humiliation and shame for many.^63,86,123^

## Misuse and misinterpretation of the concept of a functional disorder

FND undoubtedly carries with it the burden of its history and the ideas associated with it. In a modern clinical neuroscience era when it has been reformulated as a disorder of mind and brain, many clinicians still incorrectly associate the term functional to mean ‘psychological in origin’^124^ or symptoms with ‘no obvious organic origin’^125^—often with a poorly concealed view that this is an imaginary and a lesser illness. The implications are that the symptoms can be controlled, and the patient is not as deserving as those with ‘real biological’ disorders.

As long as ‘mental health issues’ or ‘psychological issues’ are artificially separated and viewed dualistically, it is likely these issues will continue to affect FND. Recognizing the stigma around mental health is a necessary part of understanding the stigma that patients with FND face. FND defies dualistic models of health and illness^126^ and is conceptualized as a disturbance in areas involved in cognitive, emotional, interoceptive and motor overlapping and interrelated domains. Moving away from outdated dichotomous models will require continuous challenging of traditional belief systems about mind, brain and body by clinicians, patients and carers in the FND community.

Some consider that using the term functional neurological disorder is an unhelpfully agnostic position, negates important psychological components and may even promote an overly ‘neurocentric’ view of the condition.^127,128^ We acknowledge that ‘functional neurological disorder’ is not a perfect term and it is likely that no term will be, given the historical and linguistic challenges. We agree with Scamvougeras and Castle,^128^ that to tackle stigma around a condition it is not enough to change a term but to challenge misconceptions and promote treatments for the condition itself. A monistic view of brain, mind and body may be most helpful when considering FND.

In the online public domain, the term ‘functional disorder’ has been widely and inaccurately depicted as a synonym for clinicians meaning ‘it’s not real’, a ‘dustbin diagnosis’ or ‘you’re imagining it’,^129^ when that is far from its intended meaning. This inaccurate misperception has extended into the conversation around other disorders, where FND symptoms may occur comorbid to the presentation, such as chronic fatigue syndrome/myalgic encephalomyelitis (CFS/ME) or long COVID syndrome.^130-133^ There have been reports of patients feeling stigma and shame when trying to obtain information about their FND diagnosis and encountering such negative material:

‘It wasn't until I started reading [online] oh my god, oh my god that's when I just lost all respect, all my self-respect … just reading things like you're nuts basically … do you know what was very unhelpful recently … from a COVID group … eh and basically it was to do with FND research being led by [name removed] basically he is a fraud, they are all frauds uhm … that just set me back just all the way’—Norah.^134^

Researching pathophysiological processes in functional disorders is essential and welcomed. Studies on structural and functional neuroimaging, the immune system, endocrine dysfunction and genetics all form part of the modern portfolio of FND research.^135-137^ However, the trend of using discoveries that are considered more biomedical in nature to ‘prove it’s a real illness, not psychological’ as is common in the long COVID and ME/CFS literature introduces a layered helping of stigma for patients and prevents an integrative and constructive approach when researching, diagnosing and treating these conditions.

## Diagnostic overshadowing: over-interpretation of investigations and under-investigation in established FND

When one form of diagnosis unduly influences the clinical assessment, it is called diagnostic overshadowing. FND is a clinical diagnosis, but people with it usually require investigations to assess for comorbid neurological and other medical conditions, which are common and easily overlooked. In this context, iatrogenic harm may result from an inappropriate diagnostic weighting or communication of investigations without considering their relevance or frequency in the general population. For example, degenerative abnormalities appear in everyone as we age such that 60% of the population over the age of 50 have ‘disc bulge’, with little evidence that these correlate at a population level to spinal pain or even predict future spinal pain.^138,139^ ‘High signal change’ and ‘white matter change’ on an MRI brain scan may be within normal limits for age but given undue weight in a clinical assessment or miscommunicated as evidence of stroke or possible MS. The mild incidental abnormality on a scan may be emphasized much more than the clinical diagnosis. EEG is often used to distinguish between epilepsy and functional seizures, but many normal EEG findings can, in some cases, be misinterpreted as epilepsy.^140^

Conversely, harm can also occur to people with established FND when they present with new symptoms and are insufficiently investigated, another consequence of diagnostic overshadowing. FND is usually a polysymptomatic disorder, and often when new symptoms arise, they are a consequence of the same condition. However, people with FND may have separate new conditions which require investigation in their own right. A balanced approach is needed not to over or under-investigate new symptoms on their own merits. The tendency for people with FND to feel that new symptoms are not being taken seriously is not unique to this condition and is experienced by people with other long-term conditions that have multiple symptoms such as MS and systemic lupus erythematosus.^141^

## Harm related to misdiagnosis of other conditions as FND and overdiagnosis of FND

The studies that we have of long-term diagnostic change in FND suggest that it is misdiagnosed no more than other neurological or psychiatric disorders.^14^ However, if there is greater awareness of the disorder, especially with a lack of understanding of how it should be diagnosed, then this may lead to an increase in misdiagnosis of people who have other neurological conditions and are wrongly told they have the condition.

Our personal experience is, over the last few years, of an increase in premature and inappropriate diagnosis of FND, especially in emergency and acute settings, by health professionals without specific neurological training. Common reasons for incorrectly diagnosing FND include assumptions made based on the presence of comorbid psychiatric disorder and making a diagnosis because tests are negative or because a symptom is unusual or not previously encountered.^142,143^

We also notice a trend for overdiagnosis in hyperacute settings, when it would be more appropriate to wait and see if symptoms become persistent, or in situations where someone has a functional neurological symptom such as mild leg weakness, which is occurring in the context of a more obvious major condition such as chronic pain, neurodevelopmental condition or a psychiatric disorder. There should be room for people to have functional neurological ‘symptoms’ as well as a ‘disorder’. Furthermore, as with any medical condition, rule in clinical signs must consider the whole presentation and its context, as opposed to blindly or in isolation—for example, motor inconsistency can be found in other disorders such as paradoxical kinesis in Parkinson’s disease.^144^

The term ‘disorder’ can in itself be harmful and unwanted by some patients, even though it is appropriate and helpful for others. Inappropriate use of the term functional neurological disorder when a more specific one, such as functional seizures or functional cognitive disorder, can also lead to harm because clinicians may assume someone has one kind of symptom when they have another. The term FND is appropriate in our view for people with severe or multiple symptoms but usually benefits from clarification of main subtype or subtypes.

## Five potential ways to address and mitigate effects of iatrogenic harm

### Reduce risk of misdiagnosis: make FND a diagnosis of inclusion

We acknowledge that misdiagnosis is an inevitable part of medicine. Some of the time, though, inappropriate diagnoses are a result of lack of knowledge and interest in FND. We are aware that in many settings, clinicians are still stuck with an outdated idea that FND needs to be diagnosed by exclusion rather than by inclusion of typical diagnostic features. Enhancing the visibility of FND at an early stage in core curricula across disciplines may help combat this. Rule in signs for functional motor symptoms include discrepancy between voluntary and automatic movements as evidenced by Hoover sign, hip abductor sign and the tremor entrainment sign, as well as functional seizure signs such as long duration of events and closed eyes during the event—there are many more rule in signs described in detail elsewhere.^2^

We recognize that some of the features of status epilepticus and functional seizures are hard to distinguish, especially in the absence of specialized training, that status epilepticus is life-threatening, and it’s rare for the above risks to be ill-intended. However, the risks could be mitigated with optimal knowledge around the semiology of differentiators between the two conditions.^42^ Maintaining a high awareness of other neurological comorbidities may help prevent some misdiagnoses. Simple educational interventions may help to reduce iatrogenic harm—one study found that rates of benzodiazepine use dropped from 78% to 41% after an educational session to emergency physicians about the features differentiating epileptic and functional seizures.^145^

It is also worth pointing out that diagnosing FND may carry a certain level of uncertainty at different points throughout the patient journey—for example, it may range from possible, probable, clinically-established, to documented. We argue this is no different to other diagnostic journeys in the field of medicine, and employing the same strategies as you would with any other disorder will help confirm if FND is present—such as comprehensive history taking, clinical examination, collateral history, video if available, investigations to look for comorbidity and follow-up in clinic to monitor illness course, while always appropriately assessing new symptoms or changes to presentation.

### Introduce FND early in the differential and contextualize investigations

Perhaps as a consequence of the idea of ‘diagnosis of exclusion’, FND is often not considered by clinicians until later in the diagnostic process. At an early stage, an open mind about the spectrum of possible diagnoses is important, but adding ‘functional’ to a list of other diagnostic categories such as ‘inflammatory, neoplastic, metabolic, genetic’ would help. Conversely, clinicians, especially more junior trainees, should not be in a rush to diagnose FND after just one meeting of the patient, especially if the symptoms are new and acute.^143^

Investigations are usually an essential part of the assessment of someone with FND, partly because neurological comorbidity is so common, and clinical evidence of comorbid conditions may be mild. The message therefore should not be to avoid investigations but do them and communicate them in a way that is least harmful to patients. Anticipating a possible ‘normal for age’ result before the investigations are done has been shown to reduce anxiety while waiting for results.^146^ More can be done to educate health professionals about the importance of casual phrases which they may consider innocuous but could have nocebo consequences like ‘wear and tear’ or ‘vascular changes in the brain’. Furthermore, in cases where there is confidence about the FND diagnosis, it has been shown that something as simple as documenting it and referring to neurology has been shown to be associated with reduced emergency department reattendance, likely reducing iatrogenic harm from multiple emergency presentations and treatments.^67^

### Teaching: with a focus on communication and stigma

There is insufficient teaching and representation of FND in core undergraduate and professional curricula.^5,104,147,148^ It would be helpful to ensure FND is a core requirement in training curricula internationally from an early stage in training, for all disciplines involved in the care of patients with FND. This has recently happened with the UK and European Association of Neurology training curricula.^149,150^ It could be worth educating professionals involved in FND formally or informally on how common stigma is for patients and the potential influence of stigma on patient outcomes.^110^ The ‘hidden curriculum’^151^ is a more subtle method of learning outside formal curricula, which includes informal conversations and role modelling—and has been found to be a means of perpetuating stereotypes but also more positive attitudes.

### Parity for FND services and research

Many healthcare professional studies have described how they feel poorly resourced to diagnose and treat patients with FND.^8,77^ People with FND are routinely excluded from both physical rehabilitation services (‘mental health problem’) and mental health services (‘neurological problem’). Achieving parity of services for FND, especially in deprived areas, would help. Treatment of FND, like many other conditions, is most beneficial when there is interdisciplinary expertise available for the patient and their family. This includes not just neurology and psychiatry but also a range of specialisms, including psychotherapy, general practitioners, occupational therapy, nursing, physiotherapy, speech and language therapy and rehabilitation medicine, among many others. Equipping all specialisms with the skills to confidently treat patients with FND will improve parity of care for this group. Further, incorporating an understanding of all aspects that influence illness, outside just the biomedical model—for example, social aspects such as environment, family dynamics and culture are important not just for FND but for all illness presentations, and such aspects could be emphasized in training. Patients with FND should be prioritized for early access to treatment (just like any other medical condition), which would go some way to reducing harm from chronicity and deconditioning, the distress of uncertainty and unnecessary investigations and treatments. On the topic of treatment, research funding for FND treatments could be improved—at the time of writing, there were less than 20 trials actively recruiting for FND compared with hundreds for epilepsy, MS and Parkinson’s disease.^152^ However, many positive things are happening with regard to research and service development, and FND is now occupying a more mainstream position in many areas.^149,153,154^

### Ongoing promotion and integration of patient and professional organizations

Patient organizations such as FND Hope (fndhope.org) and FND Action (fndaction.org.uk),^155,156^ as well as patient advocates on social media, continue to draw attention to this neglected condition, reducing stigma, challenging old paradigms and working closely with professionals and governments for improved research and service development for FND. Professional organizations and platforms are also playing a role in enhancing the visibility of FND, changing old paradigms and furthering education and research.^157,158^ Ongoing development and integration of these groups will continue to play a role in addressing some of these harms.

## Limitations

We are aware of some limitations to this review. It was not feasible to search the topic using systematic review methodology, and some of the harms we report, such as overdiagnosis of FND, arise from clinical experience rather than existing literature. It is possible that these views are not representative of other clinicians in the field. We also only included topics in the English language and so may have missed other relevant papers.

## Conclusion

Undoubtedly, there have been advances in many areas with regard to FND, and patient involvement has been integral to driving progress. That said, it continues to be the professional’s job to reduce iatrogenic harm, and more can be done to mitigate and address this issue. It is important that we continue to learn from the history of this common and treatable condition and use this knowledge in an adaptive way for the benefit of patients with FND and their carers.
